# Enhanced development of functional human NK cells in NOD‐ *scid‐IL2rg^null^* mice expressing human IL15

**DOI:** 10.1096/fj.202200045R

**Published:** 2022-08-12

**Authors:** Ken‐Edwin Aryee, Lisa M. Burzenski, Li‐Chin Yao, James G. Keck, Dale L. Greiner, Leonard D. Shultz, Michael A. Brehm

**Affiliations:** ^1^ Program in Molecular Medicine Diabetes Center of Excellence, University of Massachusetts Chan Medical School Worcester Massachusetts USA; ^2^ The Jackson Laboratory Bar Harbor Massachusetts USA; ^3^ The Jackson Laboratory Sacramento California USA

**Keywords:** Hu‐IL15, humanized mice, NK cells, NSG, transgenic

## Abstract

Human innate immunity plays a critical role in tumor surveillance and in immunoregulation within the tumor microenvironment. Natural killer (NK) cells are innate lymphoid cells that have opposing roles in the tumor microenvironment, including NK cell subsets that mediate tumor cell cytotoxicity and subsets with regulatory function that contribute to the tumor immune suppressive environment. The balance between effector and regulatory NK cell subsets has been studied extensively in murine models of cancer, but there is a paucity of models to study human NK cell function in tumorigenesis. Humanized mice are a powerful alternative to syngeneic mouse tumor models for the study of human immuno‐oncology and have proven effective tools to test immunotherapies targeting T cells. However, human NK cell development and survival in humanized NOD‐*scid‐IL2rg* ^*null*^ (NSG) mice are severely limited. To enhance NK cell development, we have developed NSG mice that constitutively expresses human Interleukin 15 (IL15), NSG‐Tg(Hu‐IL15). Following hematopoietic stem cell engraftment of NSG‐Tg(Hu‐IL15) mice, significantly higher levels of functional human CD56+ NK cells are detectable in blood and spleen, as compared to NSG mice. Hematopoietic stem cell (HSC)‐engrafted NSG‐Tg(Hu‐IL15) mice also supported the development of human CD3+ T cells, CD20+ B cells, and CD33+ myeloid cells. Moreover, the growth kinetics of a patient‐derived xenograft (PDX) melanoma were significantly delayed in HSC‐engrafted NSG‐Tg(Hu‐IL15) mice as compared to HSC‐engrafted NSG mice demonstrating that human NK cells have a key role in limiting the tumor growth. Together, these data demonstrate that HSC‐engrafted NSG‐Tg(Hu‐IL15) mice support enhanced development of functional human NK cells, which limit the growth of PDX tumors.

**Abbreviations**
FMO: fluorescence minus one
HSC: hematopoietic stem cells
IL: interleukin
IP: intraperitoneal
IV: intravenous
KIR: killer inhibitory receptors
NK: natural killer
NSG: NOD‐*scid IL2rγ* ^*null*^
PBMC: peripheral blood mononuclear cells
PBS: phosphate buffered saline
PDX: patient‐derived xenograft
Tg: transgenic
tSNE: t‐distributed stochastic neighbor embedding
UCB: umbilical cord blood

## 1 INTRODUCTION

Immunotherapy has revolutionized the treatment of human malignancies and has proven to be efficacious against a variety of cancers with improved long‐term survival rates.^1^, ^2^ However, there are groups of cancer patients that do not benefit from current immunotherapies,^3^, ^4^ leading to the challenge of testing and validating new therapies or combination of therapies that increase response rates and prolong survival in a wider range of individuals. The primary cellular mediators of immunotherapies are T cells, B cells, natural killer (NK) cells, and macrophages.^5^, ^6^, ^7^, ^8^, ^9^, ^10^, ^11^ The human innate immune system has a critical role in tumor surveillance and in immunoregulation within the tumor microenvironment.^12^ NK cells, which are naturally cytotoxic innate lymphoid cells with intrinsic anti‐tumor properties,^13^ are emerging as key targets for cancer immunotherapy.^14^, ^15^, ^16^, ^17^ NK cell function is regulated by a balance in activating and inhibitory receptor signaling.^18^ NK cells express killer inhibitory receptors (KIRs) such as CD158b, CD158e1, and killer cell lectin‐like receptor subfamily C, member 1 (CD94/NKG2A) that engage the self‐MHC class‐I and results in a negative activation signal.^19^, ^20^ Thus, cells that do not express self‐MHC class‐I are targets for NK cell killing, which is directly relevant to the control of tumors that have downregulated MHC expression. Moreover, many transformed cell populations increase the expression of stress‐induced molecules, including MHC class‐I polypeptide‐related sequence A and B (MICA and MICB). which can be recognized by activating NK cell receptors such as NKG2D.^21^ This balance of positive and negative signaling helps NK cells distinguish between transformed cells such as tumors and MHC class‐I non‐expressing cells like erythrocytes.^22^ Previous studies have demonstrated that the presence of tumor infiltrating NK cells in humans correlates with positive prognosis for multiple malignancies including ovarian carcinoma,^23^ squamous cell lung cancer,^24^ renal cell carcinoma,^25^ gastrointestinal stromal carcinoma,^26^ colorectal carcinoma,^27^ and melanoma.^28^, ^29^

Humanized mouse models that have been engrafted with functional human immune systems are being used as in vivo preclinical tools to study human immune system–tumor interactions and human‐specific therapeutics.^30^, ^31^, ^32^, ^33^ A significant limitation for use of humanized mice in cancer research had been a lack of development and survival of human NK cells after engraftment of hematopoietic stem cell (HSC) or peripheral blood mononuclear cell (PBMC), including in humanized NOD‐*scid IL2rγ* ^*null*^ (NSG) mice.^34^, ^35^ Recent studies have demonstrated several approaches to enhance the development, survival, and function of human NK cells in immunodeficient mice, including transgenic expression of cytokines,^36^, ^37^, ^38^, ^39^, ^40^, ^41^, ^42^ injection of cytokines or cytokine complexes,^43^, ^44^, ^45^, ^46^ and injection of cytokine encoding plasmids.^47^ Even with these advancements to promote NK cell development in humanized mice, there is still a significant need for preclinical models that are readily available to the research community, support engraftment of human HSC, and that do not require extensive manipulation for use. The cytokine interleukin 15 (IL15) is indispensable for the development, maturation, function, and survival of mouse and human NK cells.^48^, ^49^ Murine IL15 has limited cross‐reactivity with human, and therefore the development of human NK cells is not supported in the standard immunodeficient mouse strains used for humanization such as NSG, NOD/Shi‐*scid IL2rγ* ^*tm1Sug*^ (NOG), and BALB/c *Rag2* ^*null*^ *IL2rg* ^*null*^ (BRG). However, the expression or injection of human IL15 enables the development of human NK cells in immunodeficient mice that have been injected with human HSC.^39^, ^41^, ^44^

In this study, we describe a novel NSG‐based human IL15 transgenic mouse model. The NSG‐Tg(Hu‐IL15) mouse produces circulating human IL15 at near physiological levels.^50^ Engraftment of NSG‐Tg(Hu‐IL15) mice with human HSC results in improved and sustained NK cell numbers with enhanced maturation and functionality. Most notably, the human NK cells recovered from engrafted NSG‐Tg(Hu‐IL15) mice kill MHC class‐I deficient target cells with similar efficacy as compared to NK cells purified from human PBMC. Moreover, HSC‐engrafted NSG‐Tg(Hu‐IL15) mice show enhanced inhibition of a patient‐derived xenograft (PDX) melanoma tumor growth in an NK cell‐specific manner. These data indicate the potential utility of NSG‐Tg(Hu‐IL15) mice as an in vivo model to study human NK cell biology and as a testing platform for novel human‐specific immune‐therapeutics targeting NK cells.

## 2 MATERIALS AND METHODS

### 2.1 Mice

**FIGURE 1:**
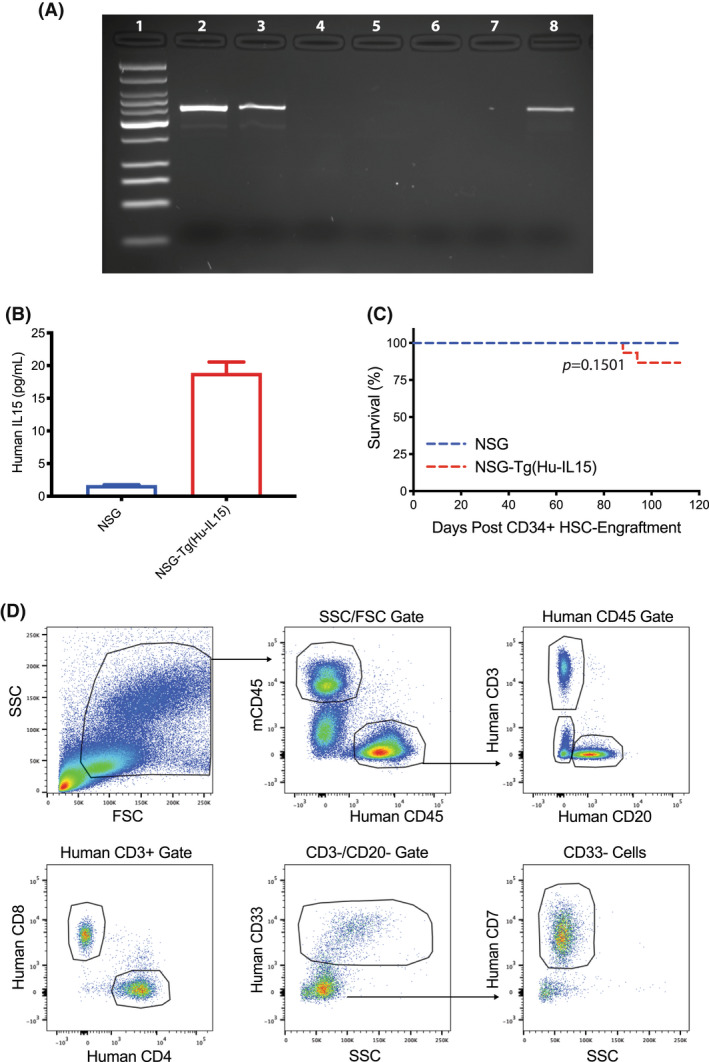
Transgenic expression of human interleukin 15 (Hu‐IL15) in NOD‐*scid IL2rγ* ^*null*^ (NSG)‐Tg(Hu‐IL15) mice. (A) Polymerase chain reactions (PCRs) from NSG‐Tg(Hu‐IL15) mice and NSG mice were visualized on an agarose gel. Lane 1, Low molecular weight DNA ladder; Lanes 2 and 3, (NSG‐Tg(Hu‐IL15) mice; Lanes 4 and 5, NSG mice; Lane 6, loading control; Lane 7, negative DNA control; and Lane 8, human IL15 DNA control. (B) Quantification of human IL15 levels in unengrafted NSG mice (*n* = 6) and homozygous NSG‐Tg(Hu‐IL15) mice (*n* = 5) of both genders that were 16 weeks of age. Human IL15 was measured in serum using ELISA and means ± SEM are shown. (C) NSG or NSG‐Tg(Hu‐IL15) mice 6 to 8 weeks of age were irradiated (200 cGy) and injected IV with 100 000 CD34+ hematopoietic stem cell (HSC) derived from human CD3‐depleted umbilical cord blood (*n* = 15 mice per group). The survival of the mice was then followed over time. (D) Representative flow data from HSC‐engrafted NSG‐Tg(Hu‐IL15) mice are shown and the gating strategy to identify human immune cell subsets is also shown. The results are representative of three independent experiments.

NOD.*Cg‐Prkdc* ^*scid*^ *Il2rg* ^*tm1Wjl*^ */SzJ* (NOD‐*scid IL2rγ* ^*null*^, NSG) mice and NOD.*Cg‐Prkdc* ^*scid*^ *Il2rg* ^*tm1Wjl*^ */SzJ Tg(IL15)SzJ* (NSG‐Tg(Hu‐IL15)) mice were obtained from colonies developed and maintained by Dr. Leonard Shultz at The Jackson Laboratory (Bar Harbor, ME). To generate NSG‐Tg(Hu‐IL15) mice, a 200Kbp BAC containing the entire human IL15 gene (RP11‐620F3) was obtained from Chori BACPAC (Emeryville, CA). Pronuclear injection of NSG embryos yielded a transgenic male founder that transmitted the human *IL15* gene to his offspring. NSG‐Tg(Hu‐IL15) hemizygous offspring were identified by polymerase chain reaction (PCR) and were used to expand the colony. NSG‐Tg(Hu‐IL15) mice were genotyped by taking tail tips (<2 mm) from weaned mice and generating DNA with a Qiagen DNEasy genomic DNA preparation kit (Qiagen USA, Germantown, MD). Specific PCR primers were designed using Primer3 (https://primer3.ut.ee/) and checked for specificity using BLAST (https://blast.ncbi.nlm.nih.gov/Blast.cgi). The forward primer is IL15F‐ACAAGGGTGATAATGCTGGC, and the reverse is IL15R‐CTGTTTCCAGTTTTTGGCAG, which together amplify a 254 base pair segment located in the 3′ end of the human IL15 gene. The primers were combined with Taq DNA polymerase (New England Biolabs Inc., Ipswich, MA), cresol red, nuclease‐free water, and deoxynucleotides, cycled on a C1000 thermal cycler (Bio‐Rad Laboratories, Inc., Hercules, California) and run out on a 2% agarose gel (See Figure 1A).

Human IL15 levels were first carried out on NSG‐Tg(Hu‐IL15) hemizygotes. Serum was collected from three female mice hemizygous for the human IL15 transgene and three female NSG non‐transgenic mice, age 55 days. The NSG‐Tg(Hu‐IL15) hemizygous mice had physiological levels of human IL15 (7.1 ± 0.3 pg/ml) while the non‐transgenic NSG mice, as expected, lacked human IL15, documenting the production of human IL15 protein. Subsequent crosses and test matings produced additional NSG‐Tg(Hu‐IL15) offspring. Genetic crosses were carried out with NSG‐Tg(Hu‐IL15) hemizygous offspring to fix the human IL15 transgene to homozygosity. NSG‐Tg(Hu‐IL15) transgenic homozygotes survived well and bred in matings of homozygotes. All animals were housed in a specific pathogen‐free facility, in microisolator cages, and given autoclaved food and maintained on sulfamethoxazole–trimethoprim medicated water (Goldline Laboratories, FL) and acidified autoclaved water on alternating weeks. All animal use was in accordance with the guidelines of the Animal Care and Use Committee of the University of Massachusetts Chan Medical School and The Jackson Laboratory and conformed to the recommendations in the *Guide for the Care and Use of Laboratory Animals* (Institute of Laboratory Animal Resources, National Research Council, National Academy of Sciences, 1996).

### 2.2 Measurement of human IL15

Human IL15 protein was measured in NSG‐Tg(Hu‐IL15) and NSG control sera by enzyme‐linked immunoassay (ELISA). NSG‐Tg(Hu‐IL15) male and female mice at 17–18 weeks of age were bled and peripheral blood was collected via submandibular puncture directly into Becton Dickinson serum separator microtainers (BD, Franklin Lakes, NJ). Samples were centrifuged, and serum was frozen at −20°C until assayed. Human IL15 levels in the sera were determined using a human IL15 Elisa Kit (BioLegend, CA). Bone marrow was flushed from the femurs of NSG‐Tg(Hu‐IL15) and NSG mice using unsupplemented Roswell Park Memorial Institute (RPMI). Bone marrow cells were cultured for 24 h in RPMI supplemented with fetal bovine serum (FBS). Supernatants were then collected, and levels of human IL15 were determined by ELISA as described above.

### 2.3 Human HSC isolation and engraftment of mice

Human umbilical cord blood (UCB) was obtained in accordance with the Committee for the Protection of Human Subjects in Research guidelines of the University of Massachusetts Chan Medical School. UCB was provided by the medical staff of the University of Massachusetts Memorial Umbilical Cord Blood Donation Program. Groups of 6‐ to 8‐week‐old male and female mice NSG and NSG‐Tg(Hu‐IL15) mice were irradiated with 200 cGy.^51^ Irradiated mice were injected IV with CD3‐depleted human UCB containing 1 × 10^5^ CD34^+^ HSC.^52^, ^53^ At the indicated time points, flow cytometry analyses of the blood of HSC recipients quantified the engraftment of the human immune system. For experimental studies, mice with >10% peripheral human CD45+ cells and >5% human CD3+ T cells were used.

### 2.4 Flow cytometry and antibodies

For analysis of human hematopoietic engraftment, the following monoclonal antibodies specific for human antigens were used: human CD45 (2D1), CD3 (UCHT1), CD4 (RPA‐T4), CD8 (RPA‐T8), CD20 (2H7) CD33 (WM53), CD7 (CD7‐6B7), CD69 (FN50) CD94 (DX22), NKp30 (P30‐15), CD158b (KIR2DL2/L3: DX27), CD56 (5.1H11), CD57 (QA17A04), CD158i (KIR2DS4: 179315), CD159c (NKG2C: 134591), CD7 (CD7‐6B7), CD314 (NKG2D: 1D11), CD159a (NKG2A: 131411), CD158e1 (KIR3DL1: DX9), CD335 (NKp46: 29A1.4), granzyme B (QA16A02), granzyme A (CB9), perforin (dG9), and CD16 (3G8). Anti‐mouse CD45 (30F‐11) was used to exclude mouse leukocytes. The antibodies were purchased from BD Biosciences, Inc (CA). or BioLegend (CA). Single‐cell suspensions of the spleens were prepared from engrafted mice, and whole blood was collected in heparin. Single‐cell suspensions of 5 × 10^5^ splenic cells in 50 μl or 100 μl of whole blood were washed with FACS buffer (phosphate‐buffered saline supplemented with 2% FBS HyClone, UT) and 0.02% sodium azide (Sigma, MO) and then pre‐incubated with rat anti‐mouse FcR11b (clone 2.4G2, BD Biosciences, CA) to block Fc binding. Specific antibodies against cell surface antigens were then added to the samples and incubated for 30 min at 4°C. Stained samples were then washed and fixed with 2% paraformaldehyde for cell suspensions or treated with BD FACS lysing solution for whole blood. For human granzymes and perforin detection, the red blood cells were lysed and the mononuclear cells fixed and then incubated with BD Cytofix/Cytoperm, Fixation/Permeabilization solution (BD biosciences, CA) for 20 min at 4°C after surface antibody staining. Cells were then stained with antibody against human granzyme A, granzyme B, and/or perforin in BD Perm/Wash buffer (BD biosciences, CA) for 30 min in the dark after washing in BD Perm/Wash buffer. For cell counts on blood, Countbright Absolute Counting Beads were used as described by the manufacturer (Thermofisher, MA). At least 100 000 events were acquired on LSRII instrument (BD Biosciences, CA) or Aurora (Cytek Biosciences, CA). Data analysis was performed using FlowJo software (Tree Star, Inc., OR). FlowJo software was used to generate t‐distributed stochastic neighbor embedding (tSNE) plots, after excluding CD3+ cells, CD20+ cells, and CD33+ cells and gating on CD7+ CD56dim CD16+ human NK cells. Human NK cells were then down‐sampled to randomly select cells from HSC‐engrafted NSG and NSG‐Tg(Hu‐IL15) mice, respectively. Down‐samples from each mouse strain were then concatenated into a single file and tSNE analysis. The results are shown in a 2D scatter plot (tSNE 2D scatter plots).

### 2.5 Chromium release assay

The NK cell cytotoxicity assay was performed as described previously.^54^, ^55^ Briefly, the target cells (human leukemia K562) were labeled with 100 μCi of 6 mCi/ml Cr‐sodium chromate (Na_2_^51^CrO_4_; Perkin‐Elmer, MA) and co‐cultured with NK effector cells isolated from human PBMCs or pooled splenocytes from HSC‐engrafted NSG‐Tg(Hu‐IL15) mice by positive selection with magnetic beads conjugated with an antibody specific for human CD56 (Miltenyi Biotec, Germany). The target cells (1 × 10^4^ cells/well) were cultured in round‐bottomed microwell plates with various concentrations of effector cells and incubated at 37°C and 5% CO_2_ for 18 h. The cells were then centrifuged at 250 *g* for 5 min and the supernatant added to Optiphase scintillation fluid (Perkin‐Elmer, MA) and incubated overnight at room temperature to allow for passive mixing and resolution of sample turbidity prior to reading. The corrected percent lysis for each concentration of effector cells was then calculated using the mean cpm for each replicate of wells: % specific lysis = 100 × [mean sample ^51^Cr‐release (cpm) − mean spontaneous ^51^Cr‐release]/[mean maximum ^51^Cr release (cpm) − mean spontaneous ^51^Cr‐release (cpm)].

### 2.6 In vivo tumor experiments and treatments

A patient melanoma tumor was obtained from the UMass Chan Medical School Cancer Avatar Institute (IRB ID: H00004721) and passaged in NSG mice to deplete the human leukocytes present within the tumor microenvironment.^56^ The PDX melanoma was then processed into ~2 × 2 × 2 mm^3^ pieces or a single‐cell suspension and either a tumor fragment or cells (2.5 × 10^6^) were transplanted subcutaneously to the right flank of human HSC‐engrafted humanized and non‐humanized NSG and NSG‐Tg(Hu‐IL15) mice.^57^ Tumor size was measured by caliper 2 to 3 times a week and volume (mm^3^) was calculated by (length × width)^2^/2. The mice were monitored for tumor growth and the indicated mice were treated with OKT8 depleting antibody (Invivogen, CA), CD335 (NKp46) depleting antibody (BAB281, Beckman Coulter, IN), or isotype control antibody (Invivogen, CA) IP at 100 μg per mouse when their tumors were between 50 and 100 mm^3^ in volume. OKT‐8 antibody and its isotype control antibody were given 3 consecutive days then every 7 days. To confirm CD8 depletion, samples from OKT‐8‐treated mice were stained with antibodies specific for human CD8β (2ST8.5H7, BD‐Bioscience). Anti‐NKp46 antibody and its isotype control were given for 2 consecutive days then every 7 days.^58^, ^59^ Tumor‐bearing mice were euthanized when tumor volumes in isotype control mice approached limits set by the Institutional Animal Care and Use Committee of UMASS Chan Medical School. The tumors, peripheral blood, and spleens were then harvested from the mice and processed for flow cytometry staining and analysis.

### 2.7 Statistical analyses

To compare individual pairwise groupings, we used unpaired t‐tests and Mann–Whitney test for parametric and nonparametric data, respectively. Three or more means were compared by one‐way ANOVA and the Bonferroni multiple comparison test. Significant differences were assumed for *p* values <.05. Statistical analyses were performed using GraphPad Prism software (version 8.0, GraphPad, CA).

## 3 RESULTS

### 3.1 Human IL15 production by NSG‐Tg(Hu‐IL15) mice

Given the importance of IL15 for NK cell development, survival, and function,^60^ levels of circulating human IL15 were measured in unengrafted NSG, and NSG mice that are homozygous (NSG‐Tg(Hu‐IL15)) for *IL15* (Figure 1). The serum human IL15 levels in 16‐week‐old NSG‐Tg(Hu‐IL15) mice were 18.8 ± 1.7 pg/ml (Figure 1B), which is comparable to physiological levels of IL15 expressed by healthy human donors.^50^ Given the potential for IL15 to be produced in the bone marrow by immune cells and stromal cells, levels of human IL15 were assessed in the bone marrow. Bone marrow was harvested from NSG and NSG‐Tg(Hu‐IL15) mice, cultured for 24 h, and human IL15 levels in the culture supernatants were determined by ELISA. Bone marrow culture supernatants from NSG‐Tg(Hu‐IL15) mice had significantly higher levels of human IL15 present than that detected from NSG mice (7.17 ± 0.46 and 0.13 ± 0.17 pg/ml, respectively; *p* < .0001). Upon engraftment with HSC, overall survival of NSG‐Tg(Hu‐IL15) mice was similar to HSC‐engrafted NSG mice (Figure 1C), indicating that the presence of the human IL15 transgene does not significantly impact the survival of NSG‐Tg(Hu‐IL15) mice upon human immune system engraftment over the 4‐month observation period.

### 3.2 Improved development of circulating human NK cells in HSC‐engrafted NSG‐Tg(Hu‐IL15) mice

**FIGURE 2:**
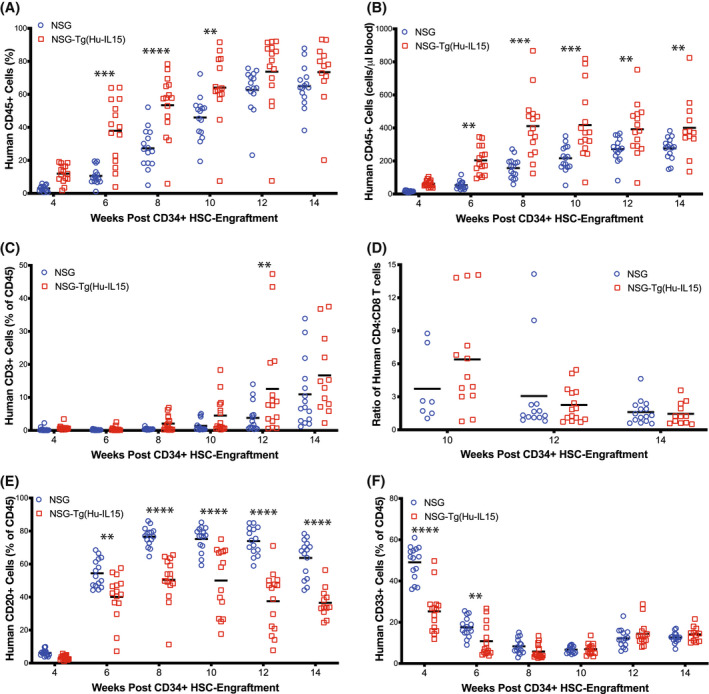
Human immune cell chimerism in NOD‐*scid IL2rγ* ^*null*^ (NSG)‐Tg(Hu‐IL15) mice. NSG (*n* = 15) or NSG‐Tg(Hu‐IL15) mice (*n* = 15) at 6 to 8 weeks of age were irradiated (200 cGy) and injected IV with 100 000 CD34+ hematopoietic stem cell (HSC) derived from human CD3‐depleted UCB as described in the Materials and Methods. Mice were bled at the indicated time points post‐injection and blood analyzed by flow cytometry for (A) frequencies of human CD45+ cells, (B) total number of human CD45+ cells per μl of blood, (C) frequencies of human CD3+ T cells, (D) ratio of CD4 to CD8 T cells, (E) frequencies of human CD20+ B cells, and (F) frequencies of human CD33+ cells. Each point represents an individual mouse. For statistical analysis, HSC‐engrafted NSG‐Tg(Hu‐IL15) mice were compared with HSC‐engrafted NSG mice; *p < .05, **p < .01, ***p < .001, ****p < .0001. The results are representative of three independent experiments.

**FIGURE 3:**
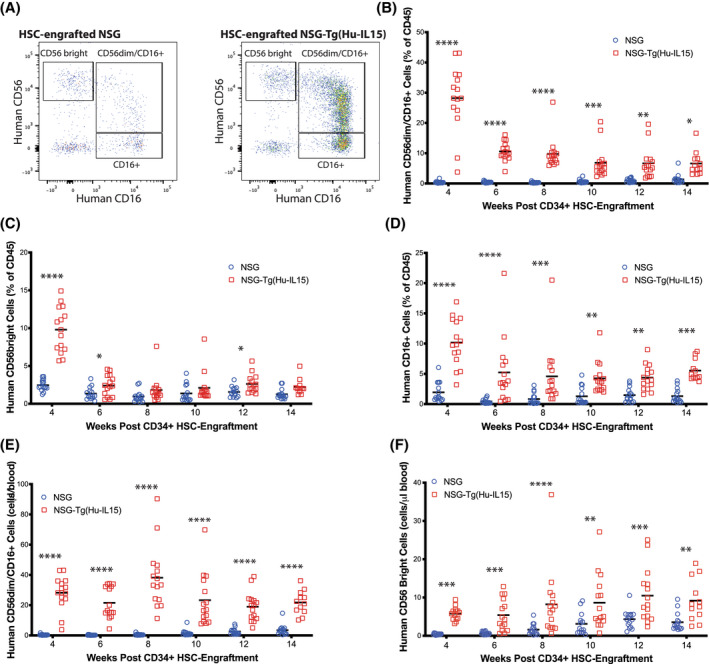
Heightened development of circulating human natural killer (NK) cells in NOD‐*scid IL2rγ* ^*null*^ (NSG)‐Tg(Hu‐IL15) mice. NSG (*n* = 15) or NSG‐Tg(Hu‐IL15) (*n* = 15) mice 6 to 8 weeks of age were irradiated (200 cGy) and injected IV with 100 000 CD34+ hematopoietic stem cell (HSC) derived from CD3‐depleted UCB as described in the Materials and Methods. Mice were bled at the indicated time points post‐injection and blood analyzed by flow cytometry for frequencies of human NK cells. (A) Representative flow plot of NK cells at 12 weeks post‐HSC injection. The human NK cells were gated on hCD45+/CD3−/CD33−/CD20−/CD7+ cells. The proportions of human (B) CD56dim/CD16+, (C) CD56bright/CD16−, and (D) CD16+ human NK cells are shown. (E) Total number of CD56dim/CD16+ NK cells per μl of blood is shown. (F) Total number of CD56‐bright NK cells per μl of blood is shown. Each point represents an individual mouse. For statistical analysis, HSC‐engrafted NSG‐Tg(Hu‐IL15) mice were compared with HSC‐engrafted NSG mice; *p < .05, **p < .01, ***p < .001, ****p < .0001. The results are representative of three independent experiments.

We next tested the impact of human IL15 expression on human immune system development over time. NSG and NSG‐Tg(HuIL15) mice were irradiated and injected IV with human HSC, and human immune system development was monitored by flow cytometry (Figures 1D–3). Figure 1D shows the representative flow cytometry data from HSC‐engrafted NSG‐Tg(Hu‐IL15) mice, and the gating strategy for the immune cell populations shown in Figure 2. HSC‐engrafted NSG‐Tg(Hu‐IL15) mice showed increased percentages of human CD45+ cell in the blood (Figure 2A) at weeks 6, 8, and 10 as compared to HSC‐engrafted NSG mice. Total numbers of human CD45+ cells were higher in HSC‐engrafted NSG‐Tg(Hu‐IL15) mice as compared to HSC‐engrafted NSG mice at 4 through 14 weeks (Figure 2B). We next compared the development of human CD3+ T cells, CD20+ B cells, CD33+ myeloid cells, and NK cells between NSG and NSG‐Tg(Hu‐IL15) mice engrafted with human HSC. Human CD3+ T cells were similar between the two strains, with slightly higher levels of T cells detected at 12 weeks in HSC‐engrafted NSG‐Tg(Hu‐IL15) mice (Figure 2C). For weeks 10, 12, and 14, the ratio of human CD4 T cells to CD8 T cells is shown. Only mice with greater than 0.5% CD3+ T cells were included in the CD4 and CD8 analysis. A higher ratio of CD4 T cells was detected for both NSG and NSG‐Tg(Hu‐IL15) mice at 10 weeks (3.7 and 6.3, respectively), 12 weeks (3.1 and 2.3, respectively), and 14 weeks (1.6 and 1.4, respectively), but there were no significant difference between the two strains. Higher percentages of human CD20+ B cells were detected in HSC‐engrafted NSG mice at 4 through 14 weeks as compared to HSC‐engrafted NSG‐Tg(Hu‐IL15) (Figure 2E). Higher percentages of human CD33+ myeloid cells were detected in HSC‐engrafted NSG mice at 4 and 6 weeks as compared to HSC‐engrafted NSG‐Tg(Hu‐IL15) (Figure 2F). Total numbers of human CD3+ T cells, CD20+ B cells and CD33+ myeloid cells are shown in Figure S1. Overall the data show that NSG‐Tg(Hu‐IL15) mice engraft with human HSC and support the development of human T cells, B cells, and myeloid cells.

Human NK cell subsets were determined by staining human CD45+ cells that were CD3−, CD20−, CD33−, and CD7+ with antibodies specific for CD56 and CD16 (Figures 1D and 3A) from weeks 4 through 14 after HSC engraftment.^61^ HSC‐engrafted NSG‐Tg(Hu‐IL15) mice showed enhanced development of human NK cell subsets in the blood as compared to HSC‐engrafted NSG mice. CD56dim/CD16+ NK cells (Figure 3B) were detected at higher percentages at all time points as compared to HSC‐engrafted NSG mice. Higher levels of CD56‐bright NK cells (Figure 3C) were detected at 4, 6, and 12 weeks in HSC‐engrafted NSG‐Tg(Hu‐IL15) mice. Higher levels of CD16+ NK cells were detected at all time points in HSC‐engrafted NSG‐Tg(Hu‐IL15) mice (Figure 3D). Total cell counts in the peripheral blood also showed a significant increase in human CD56dim/CD16+ NK cell and CD56‐bright NK cell numbers in HSC‐engrafted NSG‐Tg(Hu‐IL15) mice (Figure 3E,F, respectively), suggesting that expression of human IL15 improves the development and maintenance of human NK cell populations in HSC‐engrafted NSG mice.

### 3.3 Functional and phenotypic profiling in HSC‐engrafted NSG‐Tg(Hu‐IL15) mice

**FIGURE 4:**
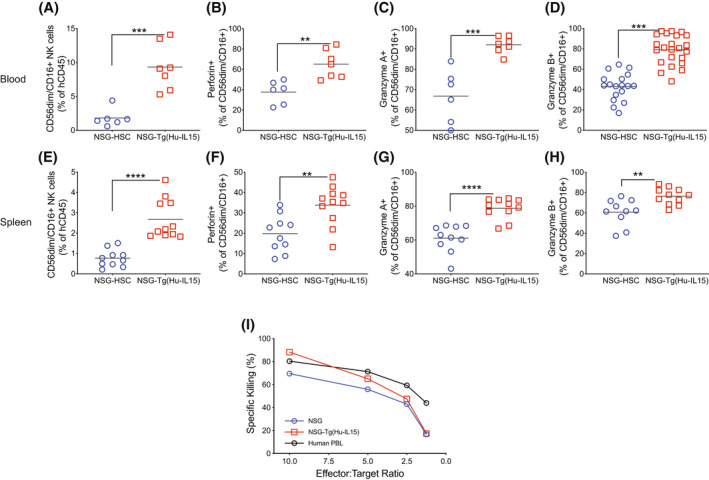
Functional profiling of natural killer (NK) cells in NOD‐*scid IL2rγ* ^*null*^ (NSG)‐Tg(Hu‐IL15) mice. NSG or NSG‐Tg(Hu‐IL15) mice 6 to 8 weeks of age were irradiated (200 cGy) and injected IV with 100 000 CD34+ hematopoietic stem cell (HSC) derived from CD3‐depleted UCB as described in the Materials and Methods. At 12 weeks post‐human HSC engraftment, blood (A, B, C, and D) and spleen (E, F, G, and H) were analyzed by flow cytometry for the frequency of (A and E) human CD56dim/CD16+ NK cells, and the proportion of human CD56dim/CD16+ NK cells to produce (B and F) perforin, (C and G) granzyme A, and (D and H) granzyme B. Each point represents an individual mouse, and the data are representative of three independent experiments. (I) Human CD56+ NK cells were purified from NSG‐Tg(Hu‐IL15) mice and from human blood and tested for their ex vivo ability to kill HLA class‐I deficient K562 tumor cells by chromium release as described in the Materials and Methods. NK cells purified from human peripheral blood mononuclear cells (PBMC) were included for comparison. The data are representative of two independent experiments. For statistical analysis, HSC‐engrafted NSG‐Tg(Hu‐Il15) mice were compared with HSC‐engrafted NSG mice; *p < .05, **p < .01, ***p < .001, ****p < .0001.

**FIGURE 5:**
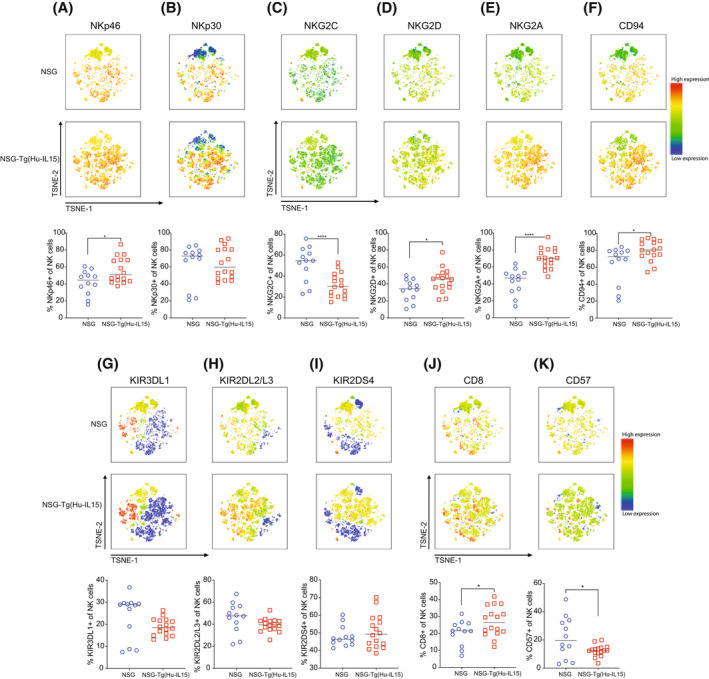
Phenotypic profiling of natural killer (NK) cells in NOD‐*scid IL2rγ* ^*null*^ (NSG)‐Tg(Hu‐IL15) mice. NSG or NSG‐Tg(Hu‐IL15) mice 6 to 8 weeks of age were irradiated (200 cGy) and injected IV with 100 000 CD34+ hematopoietic stem cell (HSC) derived from CD3‐depleted UCB as described in the Materials and Methods. At 12 weeks post‐human HSC engraftment, blood was analyzed for human CD56dim/CD16+ NK cell surface receptors by flow cytometry. Data are displayed as tSNE plots; natural cytotoxicity receptors, including NKp46 (A) and NKp30 (B); NKG family molecules, including NKG2C (C), NKG2D (D), NKG2A (E), and CD94 (F); KIRs, including KIR3DL1 (G), KIR2DL2/L3 (H), and KIR2DS4 (I); and maturation markers CD8 (J) and CD57 (K). The tSNE 2D scatter plots show the flow cytometry analysis of expression levels (red, high; blue, and low) of the surface markers. Each point represents an individual mouse. For statistical analysis, HSC‐engrafted NSG‐Tg(Hu‐IL15) mice were compared with HSC‐engrafted NSG mice; *p < .05, **p < .01, ***p < .001, ****p < .0001. The data are representative of two independent experiments.

We next examined functional and phenotypic markers expressed by human NK cells in NSG and NSG‐Tg(Hu‐IL15) mice at 12 weeks after engraftment with human HSC (Figures 4 and 5). In Figure 4, blood (Figure 4A–D) and spleen (Figure 4E–H) from HSC‐engrafted mice were analyzed by flow cytometry for the frequency of (Figure 4A,E) human CD56dim/CD16+ NK cells, and the proportion of human CD56dim/CD16+ NK cells to produce perforin (Figure 4B,F), granzyme A (Figure 4C,G), and granzyme B (Figure 4D,H). Higher percentages of CD56dim/CD16+ NK cells were detected in blood and spleen of NSG‐Tg(Hu‐IL15) mice compared to NSG mice (Figure 4A,E). A significantly higher proportion of human CD56dim/CD16+ NK cells within the blood and spleen of the NSG‐Tg(Hu‐Il15) mice stained positive for perforin (Figure 4B,F), granzyme A (Figure 4C,G), and granzyme B (Figure 4D,H) as compared to HSC‐engrafted NSG mice. Representative perforin, granzyme A, and granzyme B staining for CD56dim/CD16+ NK cells and CD56bright NK cells are shown in Supporting Information Figures S2 and S3, respectively. We next assessed the cytotoxic ability of NK cells generated within NSG‐Tg(Hu‐IL15) mice in a standard cytotoxicity assay against HLA class‐I deficient K562 cells. Human NK cells were enriched from either the spleen of HSC‐engrafted NSG mice, NSG‐Tg(Hu‐IL15) mice or human PBMC and evaluated for the ability to lyse K562 target cells using a standard Cr^51^ release assay.^55^ Human NK cells from HSC‐engrafted NSG and NSG‐Tg(hu‐IL15) mice killed the NK‐cell‐sensitive K562 cells at a similar level to NK cells isolated from human PBMC (Figure 4I). Although the levels of human NK cells in HSC‐engrafted NSG mice are significantly lower than the levels in HSC‐engrafted NSG‐Tg(Hu‐IL15) mice, the cytotoxic activity on a per cell basis is similar. Together, these data suggest that HSC‐engrafted NSG‐Tg(Hu‐IL15) mice support development of functional NK cells.

In Figure 5, blood from HSC‐engrafted mice was analyzed by flow cytometry for the proportion of human CD56dim/CD16+ NK cells expressing natural cytotoxicity receptors, including NKp46 (Figure 5A) and NKp30 (Figure 5B); NKG family molecules, including NKG2C (Figure 5C), NKG2D (Figure 5D), NKG2A (Figure 5E), and CD94 (Figure 5F); KIRs, including KIR3DL1 (Figure 5G), KIR2DL2/L3 (Figure 5H), and KIR2DS4 (Figure 5I); and maturation markers CD8 (Figure 5J) and CD57 (Figure 5K). Human CD56dim/CD16+ NK cells from HSC‐engrafted NSG‐Tg(Hu‐IL15) and HSC‐engrafted NSG mice expressed NKp46 and NKp30 (Figure 5A,B), the NKG2 family members NKG2C, NKG2D, NKG2A, and CD94 (Figure 5C–F), and KIRs (Figure 5G–I). Expression of CD8 and CD57 was also detected on CD56dim/CD16+ NK cells from HSC‐engrafted NSG‐Tg(Hu‐IL15) and HSC‐engrafted NSG mice (Figure 5J,K). While the overall expression patterns shown in Figure 5 were similar for CD56dim/CD16+ NK cells from HSC‐engrafted NSG‐Tg(Hu‐IL15) and HSC‐engrafted NSG mice, there were several statistically significant differences between the strains. A statistically significant higher proportion of NK cells from HSC‐engrafted NSG‐Tg(Hu‐IL15) mice expressed NKp46 (Figure 5A), NKG2D (Figure 5D), NKG2A (Figure 5E), CD94 (Figure 5F), and CD8 (Figure 5J) as compared to NSG mice. A statistically significant higher proportion of NK cells from HSC‐engrafted NSG mice expressed NKG2C (Figure 5C) and CD57 (Figure 5K) as compared to NSG‐Tg(Hu‐IL15) mice. Representative marker staining for CD56dim/CD16+ NK cells from HSC‐engrafted NSG‐Tg(Hu‐IL15) and HSC‐engrafted NSG mice is shown in Supporting Information Figure S4. Together, these data suggest that HSC‐engrafted NSG‐Tg(Hu‐IL15) and HSC‐engrafted NSG mice support development of human NK cells with similar expression patterns of NK‐cell‐associated phenotypic markers.

### 3.4 Growth of PDX melanoma is delayed in HSC‐engrafted NSG‐Tg(Hu‐IL15) mice

**FIGURE 6:**
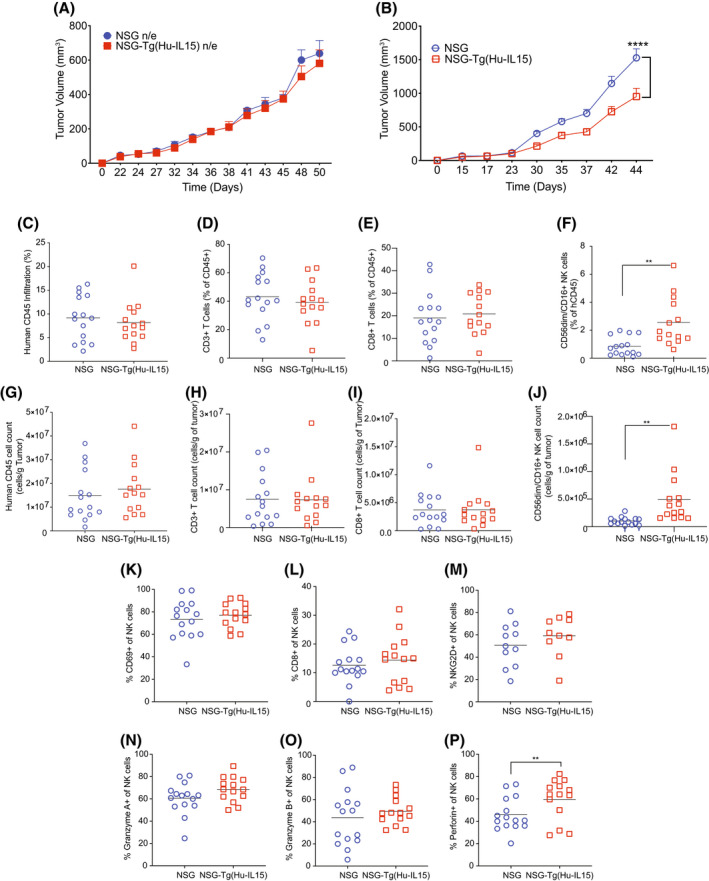
Patient‐derived xenograft (PDX) melanoma grows with reduced kinetics in hematopoietic stem cell (HSC)‐engrafted NOD‐*scid IL2rγ* ^*null*^ (NSG)‐Tg(Hu‐IL15) mice. Six to eight‐week‐old male and female NSG mice and NSG‐Tg(Hu‐IL15) mice were left unmanipulated or engrafted with human HSC were transplanted subcutaneously with PDX melanoma as described in the Materials and Methods. Tumor growth kinetics were monitored in (A) non‐engrafted (n/e, *n* = 5 mice per group) and (B) HSC‐engrafted mice (NSG, *n* = 15 and NSG‐Tg(Hu‐IL15) mice (*n* = 14). Tumors were recovered from HSC‐engrafted mice and analyzed for the percentage (C–F) and number per gram of tumor (G–J) for human CD45+ cells (C and G), human CD3+ T (D and H) cells, human CD8+ T cells (E and I) and CD56dim/CD16+ NK cells (F and J). The NK cells infiltrating the tumor microenvironment were also monitored for surface production of CD69 (K), CD8 (L), NKG2D (M) and the capacity to produce granzyme A (N), granzyme B (O) and perforin (P). Each point represents an individual animal. For statistical analysis, HSC‐engrafted NSG‐Tg(Hu‐IL15) mice were compared with HSC‐engrafted NSG mice; *p < .05, **p < .01, ***p < .001, ****p < .0001. Representative data of three independent experiments.

With the improved development and function of human NK cells in HSC‐engrafted NSG‐Tg(Hu‐IL15) mice, we next evaluated the growth kinetics for a PDX melanoma in these mice (Figure 6). We observed that tumors transplanted into unengrafted NSG‐Tg(Hu‐IL15) mice and NSG mice grew with similar kinetics (Figure 6A). However, there was a significant reduction in tumor growth kinetics in HSC‐engrafted NSG‐Tg(Hu‐IL15) mice compared to HSC‐engrafted NSG mice (Figure 6B). We interrogated the tumor microenvironment for changes in the human immune cell infiltration and phenotype by flow cytometry. No significant differences were observed in the percentages or numbers of human CD45+ cells (Figure 6C,G), human CD3+ T cells (Figure 6D,H), and human CD8+ T cells (Figure 6E,I). However, there was a significant increase in CD56dim/CD16 NK cell percentages and numbers within the tumors of HSC‐engrafted NSG‐Tg(hu‐IL15) mice compared to HSC‐engrafted NSG mice (Figure 6F,J). CD56‐bright NK cells were not detectable in the tumor microenvironment (data not shown). Similar proportions of CD56dim/CD16+ NK cells isolated from the tumor microenvironment of NSG‐Tg(Hu‐IL15) mice express functional markers CD69 (Figure 6K), CD8 (Figure 6L), and NKG2D (Figure 6M), and produce granzyme A (Figure 6N) and granzyme B (Figure 6O) when compared to NSG mice. However, a higher proportion of NK cells from the tumors in NSG‐Tg(Hu‐IL15) mice produced perforin as compared to NSG mice (Figure 6P).

### 3.5 CD8 T cells are not necessary for the reduced growth rates of the PDX melanoma in NSG Tg(Hu‐IL15)

**FIGURE 7:**
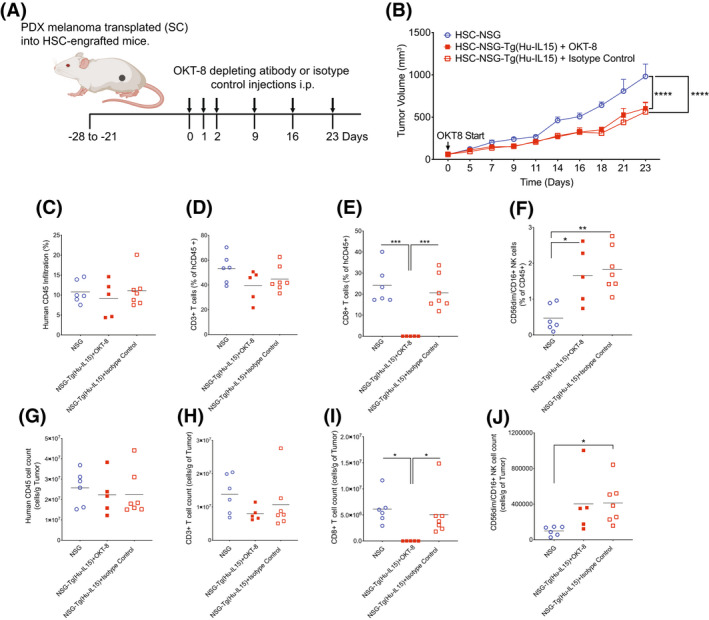
Reduced tumor growth kinetics in NOD‐*scid IL2rγ* ^*null*^ (NSG)‐Tg(Hu‐IL15) is not driven by CD8+ T cells. Hematopoietic stem cell (HSC)‐engrafted male and female NSG (*n* = 6) and NSG‐Tg(Hu‐IL15) (*n* = 12) mice were transplanted subcutaneously with patient‐derived xenograft (PDX) melanoma as described in the Materials and Methods. NSG‐Tg(Hu‐IL15) mice were then depleted of CD8+ T cells using an OKT‐8 antibody (*n* = 5) or treated with an isotype control antibody (*n* = 7). (A) Experimental design and treatment schedule for OKT‐8 depletion of CD8 T cells. (B) Tumor growth kinetics were then monitored in the mice. The tumors were harvested from the mice and analyzed for the percentage (C–F) and number per gram of tumor (G–J) for human CD45+ cells (C and G), human CD3+ T (D and H) cells, human CD8+ T cells (E and I), and CD56dim/CD16+ NK cells (F and J). Each point represents an individual animal. For statistical analysis, CD8+ T cell‐depleted NSG‐Tg(Hu‐Il15) mice were compared with NSG‐Tg(Hu‐IL15) and NSG mice; *p < .05, **p < .01, ***p < .001, ****p < .0001. Representative data of two independent experiments.

IL15 is important for NK cell development and function and CD8+ T‐cell memory maintenance.^62^ We next investigated the potential role of CD8+ T cells in the reduced rate of tumor growth in NSG‐Tg(hu‐IL15) mice (Figure 7A). HSC‐engrafted NSG‐Tg(Hu‐IL15) and NSG mice were implanted subcutaneously with 2.5 million PDX melanoma cells. When the tumor became palpable, CD8+ T cells were depleted from half of the NSG‐Tg(Hu‐IL15) mice using OKT‐8 depleting antibody as described in the Materials and Method. Depletion of CD8+ T cells in the PDX melanoma bearing NSG‐Tg(Hu‐IL15) mice did not significantly change the tumor growth rates as compared to isotype control treated HSC‐engrafted NSG‐Tg(Hu‐IL15) mice (Figure 7B). Infiltrating human CD45+ cells and CD3+ T cells were detectable in the tumors of mice treated with OKT‐8 (Figure 7C,D,G,H). However, human CD8 T cells were not detectable after staining with an antibody specific for CD8β, confirming CD8 T‐cell depletion in OKT‐8‐treated mice (Figure 7E,I). Within the tumor microenvironment, HSC‐engrafted NSG‐Tg(Hu‐IL15) mice maintained higher CD56dim/CD16+ NK cell percentages and numbers as compared to tumors from HSC‐engrafted NSG mice after CD8+ T‐cell depletion (Figure 7F,J). These data suggest that human CD8 T cells do not contribute to the reduced growth kinetics of the PDX melanoma in HSC‐engrafted NSG‐Tg(Hu‐IL15) mice as compared to NSG mice.

### 3.6 Depletion of human NK cells from HSC‐engrafted NSG‐Tg(Hu‐IL15) mice results in faster growth kinetics for the PDX melanoma

**FIGURE 8:**
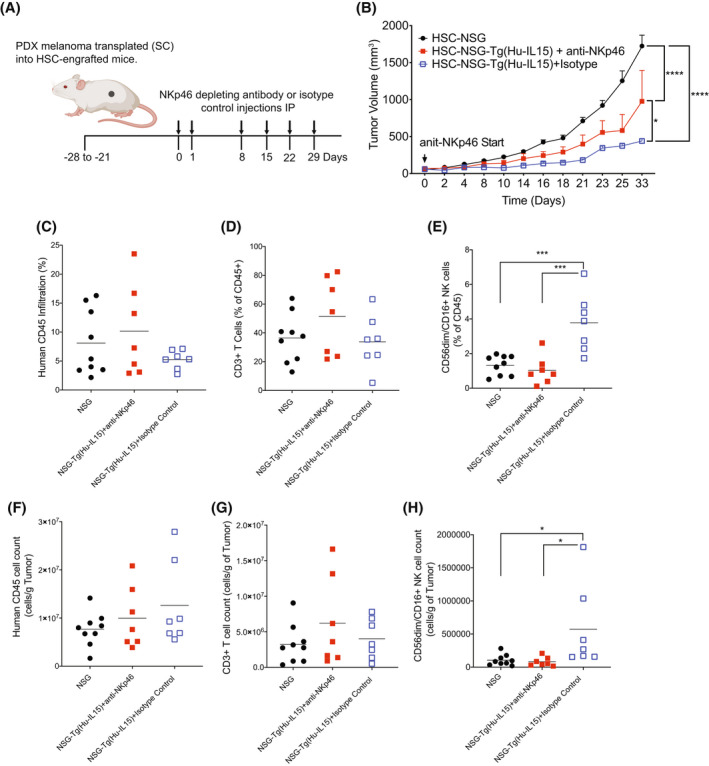
Reduced tumor growth kinetics in NOD‐*scid IL2rγ* ^*null*^ (NSG)‐Tg(Hu‐IL15) partially restored by the depletion of NKp46+ NK cells. Hematopoietic stem cell (HSC)‐engrafted male and female NSG (*n* = 9) and NSG‐Tg(Hu‐IL15) mice (*n* = 14) were transplanted subcutaneously with patient‐derived xenograft (PDX) melanoma as described in the Materials and Methods. NSG‐Tg(Hu‐IL15) mice were then depleted of natural killer (NK)p46+ NK cells using an NKp46 depleting antibody (*n* = 7) or treated with an isotype control antibody (*n* = 7). (A) Experimental design and treatment schedule for NK cell depletion. (B) Tumor growth kinetics were then monitored in the mice. The tumors were harvested from the mice and analyzed for the percentage (C–E) and number per gram of tumor (F–H) of human CD45+ cells (C and F), human CD3+ T cells (D and G), and CD56dim/CD16+ NK cells (E and H). Each point represents an individual animal. For statistical analysis, NKp46+ NK cell‐depleted NSG‐Tg(Hu‐IL15) mice were compared with NSG‐Tg(Hu‐IL15) and NSG mice; *p < .05, **p < .01, ***p < .001, ****p < .0001. Representative data of two experiments.

To determine whether human NK cells mediated the delay in PDX melanoma growth kinetics observed in NSG‐Tg(Hu‐IL15) mice, NK cells were depleted using a NKp46 depleting antibody as previously described.^58^, ^59^ In HSC‐engrafted NSG‐Tg(Hu‐IL15) mice, approximately 50%–80% of CD56dim/CD16+ NK cells are brightly expressing NKp46 (Figure 5A), and therefore treatment with the NKp46 depleting antibody is predicted to deplete a large number of the human NK cells. Tumor growth kinetics were compared to HSC‐engrafted NSG‐Tg(Hu‐IL15) and NSG mice that were not treated with the anti‐NKp46 antibody (Figure 8). HSC‐engrafted NSG‐Tg(Hu‐IL15) mice that were treated with the NKp46 depleting antibody showed increased tumor growth kinetics as compared to isotype treated HSC‐engrafted NSG‐Tg(Hu‐IL15) mice (Figure 8B). Within the tumor microenvironment, the depletion of NK cells did not affect the percentages and numbers of human CD45+ cells (Figure 8C,F) and human CD3+ T cells (Figure 8D,G). The depletion however lowered the percentage and number of CD56dim/CD16+ NK cells within the tumor microenvironment to levels similar to those in NSG mice (Figure 8E,H), suggesting that tumor killing in NSG‐Tg(hu‐IL15) mice was a result of increased NK cell numbers and function.

## 4 DISCUSSION

As the utility of the immunodeficient mouse strains used to generate humanized mice continue to advance, these models are becoming invaluable preclinical tools for biomedical research, and are increasingly useful for immuno‐oncology both in drug development and immunotherapy assessment.^1^, ^30^ Although the genomic editing of humanized mice has become progressively more sophisticated, the current iteration of human immune system bearing mice based on the NSG strain shows low frequencies and impaired functional development of circulating human NK cells.^34^ The lack of efficient human NK cell development in NSG mice was hypothesized to be a result of deficiency of human IL15, which is required for human NK cell development and a lack of species cross‐reactivity for mouse IL15.^46^ We tested this hypothesis by the transgenic expression of human IL15 in NSG mice. The transgene, generated using a BAC containing the human *IL15* gene driven by the human IL15 promoter, leads to the expression of circulating human IL15 at 18.8 ± 1.7 pg/ml. Numerous studies have measured the levels of human monomeric IL15 in the serum of healthy donors and patients with underlying disease. These studies agree that for most healthy individuals, the levels of monomeric IL15 are within an interquartile range of 0–8.68 pg/ml, with a median value of 0.83 pg/ml.^50^, ^63^, ^64^, ^65^ Moreover, levels of human IL15 above 20 pg/ml are often associated with inflammatory disease.^66^ Together, these results suggest that the levels of human IL15 detected in the serum of NSG‐Tg(Hu‐IL15) mice are comparable to levels in healthy donors and lower than levels found in donors with inflammatory disease. NSG‐Tg(Hu‐IL15) mice engraft with human HSC show overall good survival post‐engraftment and support the enhanced development of human NK cells.

IL15 is an important a key regulator of NK cell biology, including their generation, maintenance, and function.^67^, ^68^ The IL15 receptor is comprised of the IL2rβ and IL2rγ chains that are assembled on the surface of the NK cell progenitors that bind IL15 complexed with the IL15α chain presented in a trans or cis manner.^69^, ^70^ Loss of IL15 in mice leads to severe reduction in NK cell numbers.^71^ The administration of recombinant human IL15‐IL15α complexes into BRG mice and expression of human IL15 in BRG mice expressing human SIRPα (SRG‐15) that are engrafted with human HSC led to a significantly improved human NK cell development and/or survival.^39^, ^41^, ^44^, ^46^, ^72^ In addition, transgenic expression of human IL15 in NOG mice supports the in vivo survival of human NK cells enriched from peripheral blood samples.^40^, ^73^ HSC‐engrafted NSG‐Tg(Hu‐IL15) mice showed heightened NK cell frequency in the periphery and tissues that occurred very early post‐reconstitution and was sustained throughout the 14‐week observation period. In line with enhanced maturation and function within HSC‐engrafted NSG‐Tg(Hu‐IL15) mice, the circulating and splenic NK cells had better capacity to produce granzymes and perforin and increased expression of multiple phenotypic and functional markers including the activation receptors NKp46, NKG2D, and CD94 than those generated in non‐transgenic NSG mice. We however observed the upregulation of the inhibitory receptor NKG2A and downregulation of the activation receptor NKG2C. NSG‐Tg(Hu‐IL15) mice also support the transient survival of human NK cells purified from peripheral blood at higher levels as compared to NSG mice (data not shown), and additional experiments are underway characterize these cells.

Previous studies have shown that sustained stimulation with high concentrations of IL15 in vivo results in the accumulation of mature mouse or human NK cells with impaired functionality and an altered balance in activation and inhibitory receptors.^74^, ^75^ Certainly, in NOG‐IL15 Tg mice, altered phenotypic receptor expression and reduction of in‐vitro cytotoxicity activity were observed.^40^ In HSC‐engrafted NSG‐Tg(Hu‐IL15) mice, human NK cells have an improved capacity for cytotoxic activity with the observed improvement in perforin and granzyme A/B production. Moreover, human NK cells from NSG‐Tg(Hu‐IL15) were able to ex vivo kill K562 cells with similar efficiency as those from human PBMC. HSC reconstituted NSG‐Tg(Hu‐IL15) mice also significantly impaired the growth of a PDX melanoma compared with NSG mice. Together, these data suggest that while the sustained expression of human IL15 may be impacting the phenotypic receptor profile, the effect is not pronounced enough to push the human NK cells toward senescence and/or anergy with impaired functionality. We believe this observed difference in transgene behavior could be due to the near physiological levels of human IL15 produced in the NSG‐Tg (Hu‐IL15) mice.

Our results indicate that CD56dim/CD16+ NK cells from HSC‐engrafted NSG‐Tg(Hu‐IL15) mice appear phenotypically and functionally mature by many measures, but there are still differences between these cells and the NK cells in humans. For example, CD57 is expressed by 30%–60% of the CD56dim/CD16+ NK cells found in human blood,^76^ while less than 20% of CD56dim/CD16+ NK cells in the blood of NSG‐engrafted NSG‐Tg(Hu‐IL15) were positive for CD57 expression. NK cells expressing CD57 have a higher capacity for cytotoxicity and expression of cytokines following engagement of CD16 but have a reduced capacity for proliferation, suggesting that CD57+ NK cells may be terminally differentiated.^77^ In addition, the number of NK cells expressing CD57 in human blood increases with age.^76^ Overall these findings suggest that there are maturation differences between CD56dim/CD16+ NK cells from humans and HSC‐engrafted NSG‐Tg(Hu‐IL15) mice and that CD57 expression may change over time in HSC‐engrafted NSG‐Tg(Hu‐IL15) mice. Studies are currently underway to assess the functionality of CD57+ and CD57‐ NK cells in HSC‐engrafted NSG‐Tg(Hu‐IL15) mice over time.

We also show that NK cells generated in HSC‐engrafted NSG‐Tg(Hu‐IL15) mice infiltrated the tumor microenvironment at a significantly higher level than in tumor‐bearing NSG non‐transgenic mice. Additionally, the observed reduction in tumor growth was driven primarily by NK cells and not CD8 T cells, which corresponds to the enhanced NK cell development, maturation, and function in NSG‐Tg(Hu‐IL15) mice. The observed lack of effect on tumor growth with the depletion of CD8 T cells was unexpected but not surprising. Although they are important for tumor immune surveillance, the CD8 T cells within the TME can be dysfunctional.^78^ Also, while the presence of circulating IL15 in humanized mice has the potential of improving CD8 memory T‐cell development and functional maturation as observed in HSC‐engrafted SRG‐15 mice, we did not observe any change in CD8 T cell phenotype (data not shown) and therefore did not expect improvement in CD8 T‐cell function within the TME.^39^ The development of functional NK cells within HSC‐engrafted NSG‐Tg(Hu‐IL15) mice creates a platform within immuno‐oncology for the in vivo study of NK cells in tumor–immune system interactions and testing novel immunotherapies mediated by NK cells. The reduction in tumor growth though significant was not as robust as would have been expected which points to potential NK cell dysfunction within the TME. The NSG‐Tg(Hu‐IL15) mouse may therefore provide a potential model for boosting NK cell function within tumors.

The development of NK cell‐mediated immune therapies will benefit from an in vivo model that could clarify their mechanisms of action and evaluate their efficacy before advancement to clinical trial. Moreover, an effective humanized model for personalized medicine will help interrogate the benefit of specific NK‐cell‐based treatment for patient‐specific malignancies. For example, approximately 21% of primary melanoma and 44% of metastatic melanoma samples lack expression of HLA class‐I antigen, an important component for CD8+ T‐cell‐mediated therapies.^79^ Additionally, check point inhibition‐based therapies targeting PD1‐PD‐L1 interactions have a durable response rate of 20%–50% in metastatic melanoma,^80^ potentially due to acquired resistance by loss of HLA class‐I.^81^, ^82^ NK cells are therefore appealing candidates for HLA class‐I deficient melanomas.^83^, ^84^, ^85^ In summary, we have developed a novel NSG mouse expressing human IL15 at physiological levels that enhances human NK cell development and function, creating a platform for the in vivo study of human NK cell biology and for the development and preclinical evaluation of NK cell targeting immunotherapies.

## AUTHOR CONTRIBUTIONS

Ken‐Edwin Aryee, Dale L. Greiner, Leonard D. Shultz, and Michael A. Brehm conceived, designed, and performed the project. Ken‐Edwin Aryee, Lisa M. Burzenski, and Li‐Chin Yao performed the animal experiments, sample collection, and laboratory experiments and analyzed the data. Ken‐Edwin Aryee, Dale L. Greiner, Leonard D. Shultz, and Michael A. Brehm wrote the manuscript. Lisa M. Burzenski, Li‐Chin Yao, and James G. Keck reviewed the manuscript. All authors contributed to the article and approved the submitted version.

## DISCLOSURES

The contents of this publication are solely the responsibility of the authors and do not necessarily represent the official views of the National Institutes of Health. MAB and DLG receive research support and are consultants for The Jackson Laboratory.

## ETHICS STATEMENT

The animal studies were reviewed and approved by the Animal Care and Use Committee of the University of Massachusetts Chan Medical School and The Jackson Laboratory. The studies using human tissues were reviewed and approved by the Institutional Review Board of the University of Massachusetts Chan Medical School.

## Supporting information


Appendix S1

## Data Availability

The raw data supporting the conclusions of this article will be made available by the authors, without undue reservation.
