# Supplementary material for: Enhanced development of functional human NK cells in NOD‐ scid‐IL2rgnull mice expressing human IL15
Source: FASEB J. 2022 Aug 12;36(9):e22476. doi: 10.1096/fj.202200045R (PMC9383543; doi:10.1096/fj.202200045R)
Supplement: Supplementary file 1 — Appendix S1 [file FSB2-36-0-s001.docx]

Supplemental Figure 1

**Supplemental Figure 1. Total number of human T cells, B cells and myeloid cells in HSC-engrafted NSG-Tg(Hu-IL15) mice.** NSG (n=15) or NSG-Tg(Hu-IL15) mice (n=15) at 6 to 8 weeks of age were irradiated (200 cGy) and injected IV with 100,000 CD34+ HSC derived from human CD3-depleted UCB as described in the Materials and Methods. Mice were bled at the indicated time points post injection and blood analyzed by flow cytometry for (A) total number of human CD3+ T cells per μl of blood, (B) total number of human CD20+ B cells per μl of blood, and (C) total number of human CD33+ myeloid cells per μl of blood. Each point represents an individual mouse. For statistical analysis HSC engrafted NSG-Tg(Hu-IL15) mice were compared with HSC-engrafted NSG mice; * p<0.05, *** p<0.001, **** p<0.0001. The results are representative of 3 independent experiments.

Supplemental Figure 2

**Supplemental Figure 2. Perforin, granzyme A and granzyme B representative flow cytometry stains for CD56dim/CD16+ human NK cells**. NSG or NSG-Tg(Hu-IL15) mice 6 to 8 weeks of age were irradiated (200 cGy) and injected IV with 100,000 CD34+ HSC derived from CD3-depleted UCB as described in the Materials and Methods. At 12 weeks post human HSC engraftment, human CD56dim/CD16+ NK cells from blood (A to I) and spleen (J to R) were analyzed by flow cytometry for the production of perforin (A, D, G, J. M. P), granzyme A (B, E, H, K, N, Q), and granzyme B (C, F, I, L, O, R). FMO samples were pools of blood or spleen from both mouse strains.

Supplemental Figure 3

**Supplemental Figure 3. Perforin, granzyme A and granzyme B representative flow cytometry stains for CD56bright human NK cells**. NSG or NSG-Tg(Hu-IL15) mice 6 to 8 weeks of age were irradiated (200 cGy) and injected IV with 100,000 CD34+ HSC derived from CD3-depleted UCB as described in the Materials and Methods. At 12 weeks post human HSC engraftment, human CD56bright NK cells from blood (A to I) and spleen (J to R) were analyzed by flow cytometry for the production of perforin (A, D, G, J. M. P), granzyme A (B, E, H, K, N, Q), and granzyme B (C, F, I, L, O, R). FMO samples were pools of blood or spleen from both mouse strains.

Supplemental Figure 4

**Supplemental Figure 4. Representative flow cytometry stains for phenotypic profiling of CD56dim/CD16 human NK cells**. NSG or NSG-Tg(Hu-IL15) mice 6 to 8 weeks of age were irradiated (200 cGy) and injected IV with 100,000 CD34+ HSC derived from CD3-depleted UCB as described in the Materials and Methods. At 12 weeks post human HSC engraftment, blood was analyzed for human CD56dim/CD16+ NK cell surface receptors by flow cytometry. Representative flow cytometry data are shown for; natural cytotoxicity receptors, including NKp46 (A) and NKp30 (B); NKG family molecules, including NKG2C (C), NKG2D (D), NKG2A (E) and CD94 (F); killer cell immunoglobulin-like receptors, including KIR3DL1 (G), KIR2DL2/L3 (H) and KIR2DS4 (I); and maturation marker CD57 (J).
